# Outcomes of Intravitreal Anti-VEGF Therapy for Neovascular Age-Related Macular Degeneration in Kazakhstan

**DOI:** 10.3390/vision10040082

**Published:** 2026-10-14

**Authors:** Marat Suleimenov, Baurzhan Kadyrgaliyev, Elmira Kanafyanova, Zhanar Meyermanova, Venera Abdullina, Zulfiya Zhankalova, Ainur Yeshmanova, Akmaral Abdykulova, Svetlana Almukhambetova, Mukhit Kulmaganbetov

**Affiliations:** 1 Department of Ophthalmology, Asfendiyarov Kazakh National Medical University, Almaty 050012, Kazakhstan; suleymenov.m@kaznmu.kz (M.S.); kadyrgaliyev.b@kaznmu.kz (B.K.);; 2 Science Management Department, Kazakh Eye Research Institute, Almaty 050012, Kazakhstan; 3 Department of General Medical Practice №1, Asfendiyarov Kazakh National Medical University, Almaty 050012, Kazakhstan; 4 Department of Molecular Biology and Medical Genetics, Asfendiyarov Kazakh National Medical University, Almaty 050012, Kazakhstan; almukhambetova.s@kaznmu.kz; 5 Centre for Sanitary and Epidemiological Expertise of the Medical Centre of the Presidential Administration of the Republic of Kazakhstan, Astana 010020, Kazakhstan; 6 Quantum Optics Lab, Centre for Eye and Vision Research (CEVR), Hong Kong SAR, China

**Keywords:** age-related macular degeneration, anti-VEGF, central retinal thickness, best recorded visual acuity, photodynamic therapy

## Abstract

Background: Neovascular age-related macular degeneration (nAMD) is a leading cause of irreversible vision loss among older adults. Although randomised controlled trials have demonstrated the robust efficacy of intravitreal anti-vascular endothelial growth factor (anti-VEGF) therapy, real-world outcomes often differ markedly from trial findings. This study aimed to evaluate the anatomical and functional outcomes of anti-VEGF therapy in a clinical cohort of nAMD patients, including a subgroup that received adjunctive rescue photodynamic therapy (PDT) with verteporfin. Methods: This single-centre, retrospective observational cohort study included 568 patients (634 eyes) with nAMD treated with intravitreal anti-VEGF injections. Bevacizumab was used in 49% of eyes, ranibizumab in 44%, and aflibercept in 7%. Best recorded visual acuity (BRVA) and central retinal thickness (CRT) measured by optical coherence tomography (OCT) were recorded at baseline and at the final documented follow-up. Eyes with eccentric fixation were excluded from BRVA analysis but were retained for CRT analysis. Results: Mean patient age was 71.7 ± 8.8 years, and 67.6% of eyes were from female patients. The mean number of anti-VEGF injections was 5 ± 5.3 per eye; the mean follow-up duration was 11.5 months. Mean CRT decreased significantly from 347.3 ± 27.5 μm at baseline to 324.9 ± 28.8 μm at final follow-up (*p* < 0.001). Mean BRVA (logMAR) improved modestly from 0.98 ± 0.55 (Snellen ~20/190; ~35 ETDRS letters) to 0.93 ± 0.56 (Snellen ~20/175; ~37.5 ETDRS letters) (*p* < 0.001), with 67.9% of eyes demonstrating BRVA stability (change < 0.1 logMAR). Rescue PDT was administered in 10.3% of eyes. Conclusions: In this real-world cohort, anti-VEGF therapy was associated with significant anatomical improvement, as measured by CRT reduction, while functional outcomes were characterised by visual stabilisation rather than substantial gains.

## 1. Introduction

Neovascular age-related macular degeneration (nAMD) is characterised by the growth of abnormal choroidal neovascular membranes beneath the retinal pigment epithelium and neurosensory retina [1], leading to exudation, haemorrhage, and ultimately irreversible central vision loss [1,2,3]. It remains one of the principal causes of severe visual impairment in individuals over the age of 60 years globally, with its prevalence expected to rise substantially as populations age [4,5]. The introduction of intravitreal anti-vascular endothelial growth factor (anti-VEGF) agents, including bevacizumab, ranibizumab, and aflibercept, has fundamentally transformed the therapeutic landscape for nAMD [6], offering the possibility not only of halting disease progression but also of achieving meaningful visual improvement.

Landmark randomised controlled trials (RCTs) have established the efficacy of anti-VEGF therapy with remarkable consistency [7,8,9]. The Comparison of Age-Related Macular Degeneration Treatments Trials (CATT) demonstrated that ranibizumab and bevacizumab produced equivalent visual acuity gains of approximately 8 letters at one year when administered monthly [10,11]. The VIEW 1 and VIEW 2 trials showed that aflibercept dosed every 2 months after three monthly loading doses was non-inferior to monthly ranibizumab, with mean gains of 7.8 to 8.1 letters [12]. Similarly, the MARINA and ANCHOR trials reported mean gains of 6.6 to 11.3 Early Treatment Diabetic Retinopathy Study (ETDRS) letters with ranibizumab [13,14,15,16], establishing a strong evidence base for anti-VEGF therapy for nAMD.

Despite these compelling RCT results, a substantial and well-documented gap exists between the efficacy observed in clinical trials and the effectiveness achieved in routine clinical practice. Multiple real-world studies and registries, including the AURA study and “Fight Retinal Blindness!” registry [17,18], have consistently reported smaller visual acuity gains in clinical settings, typically ranging from 0 to 5 ETDRS letters. This discrepancy has been attributed to several factors, including less frequent monitoring and treatment (under-treatment relative to trial protocols), late presentation with established disease and poor baseline visual acuity, higher rates of treatment non-adherence, and comorbidities that limit visual potential [19,20,21,22]. Understanding the magnitude and drivers of this efficacy–effectiveness gap is critical for optimising real-world nAMD management and setting realistic expectations for patients and clinicians.

The primary objective of this study was to evaluate the real-world structural and functional outcomes of intravitreal anti-VEGF therapy in a large single-centre retrospective cohort of patients with nAMD. A secondary objective was to describe the anatomical and functional outcomes in a subgroup of eyes that required adjunctive rescue photodynamic therapy (PDT) with verteporfin for persistent or recurrent exudation despite anti-VEGF treatment. By analysing a dataset derived from routine clinical practice, this study aims to contribute to the growing body of real-world evidence on nAMD management outcomes.

## 2. Materials and Methods

### 2.1. Study Design and Setting

This was a single-centre, retrospective observational cohort study conducted in the Department of Ophthalmology at Asfendiyarov Kazakh National Medical University (Almaty, Kazakhstan). The study was performed in accordance with the tenets of the Declaration of Helsinki and was approved by the Local Ethics Committee of the Asfendiyarov Kazakh National Medical University (Code: 3-126, Date: 30 March 2022). Given the retrospective design and use of de-identified clinical data, the requirement for informed consent was waived. Consecutive patients diagnosed with neovascular AMD who received at least one intravitreal anti-VEGF injection between the database inception and the data collection endpoint were included in the study. The anti-VEGF agents administered included bevacizumab, ranibizumab, and aflibercept, with the specific agent and treatment regimen determined at the discretion of the treating physician based on clinical judgment, drug availability, and patient preference.

### 2.2. Participants

Inclusion criteria were (1) a diagnosis of neovascular (exudative) age-related macular degeneration confirmed by clinical examination and spectral-domain optical coherence tomography (SD-OCT); (2) availability of baseline best recorded visual acuity (BRVA) and central retinal thickness (CRT) measurements prior to the first anti-VEGF injection; (3) at least one documented follow-up visit with BRVA and CRT measurements after anti-VEGF treatment; and (4) treatment with intravitreal anti-VEGF therapy (bevacizumab, ranibizumab, or aflibercept). ‘Baseline’ was defined as the visit immediately preceding the first anti-VEGF injection at the department. All eyes were treatment-naïve. Patients were excluded if they had insufficient data for either baseline or follow-up measurements. There was no minimum or maximum follow-up duration specified, as the study aimed to capture real-world practice patterns where follow-up intervals are variable.

Data were extracted from an institutional clinical database. The following variables were recorded: patient identifier, gender, age at presentation, laterality of the affected eye(s), number of anti-VEGF injections administered, BRVA (recorded in decimal notation) before and after treatment, and CRT (average thickness of the central 1-mm diameter subfield) before and after treatment. OCT imaging was performed using a Zeiss Cirrus 6000 (Carl Zeiss Meditec AG, Jena, Germany). The 6 × 6 mm macular cube (512 a-scans making 128 b-scans) was used. Several data cleaning rules were applied to ensure analytical integrity. Eyes that switched anti-VEGF agents during the follow-up period (4% of cases) were analysed according to the initial agent received to maintain an intention-to-treat framework for agent-specific subgroups.

Most entries in the database represented a single eye (OD for right eye, OS for left eye). However, some entries were labelled “OU” (both eyes) and contained paired data formatted as “OD = [value] OS = [value]” in the BRVA and CRT columns. For the purposes of statistical analysis, these OU entries were parsed into individual eye-level observations, with the OD and OS values extracted individually, and analysed using a patient-level mixed-effects model to account for inter-eye correlation. This approach yielded a total analytic sample of 634 eyes from 568 unique patients. Eyes were categorised into two subgroups: “Anti-VEGF Monotherapy” and “Anti-VEGF + Rescue PDT”.

Visual acuity was recorded using a Snellen chart at a standard testing distance of 5 m. As formal refraction was not performed at every routine follow-up visit, pinhole correction was used when central fixation allowed. Therefore, visual acuity is reported as best recorded visual acuity (BRVA) rather than best-corrected visual acuity. Some BRVA entries contained qualitative descriptors such as eccentric fixation. These text-annotated entries indicate that the patient could not achieve central fixation, rendering standard decimal BRVA measurements non-quantifiable in a comparable manner. A total of 28 eyes with eccentric fixation were excluded from all numerical BRVA statistical analyses but were retained in the CRT anatomical analysis. Additionally, one eye was noted as having no light perception and was similarly excluded from BRVA analysis. BRVA values were converted from decimal notation to logMAR for statistical testing using the formula: logMAR = −log10(decimal BRVA). For low vision levels that could not be expressed as standard decimals, counting fingers (CF) was assigned a logMAR value of 1.85 and hand movements (HM) a value of 2.3.

### 2.3. Outcome Measures

The primary anatomical outcome was the change in CRT from baseline to the final documented follow-up, as measured by SD-OCT. The primary functional outcome was the change in BRVA from baseline to the final documented follow-up. Visual stability was defined as a change < 0.1 logMAR. Clinically significant improvement or worsening were defined as a change ≥ 0.3 logMAR. Secondary outcomes included the proportion of eyes gaining or losing 1 or more ETDRS lines (defined as a change of 0.1 logMAR or greater), the total number of anti-VEGF injections administered, and the proportion of eyes requiring adjunctive rescue PDT.

### 2.4. Statistical Analysis

Descriptive statistics were used to summarise baseline demographic and clinical characteristics. Continuous variables were reported as mean ± standard deviation (SD) or median with interquartile range and 95% confidence intervals (CIs), where appropriate. Categorical variables were reported as counts and percentages. Given that preliminary assessment revealed non-normal distributions for both CRT and BRVA change values (as assessed by the Shapiro–Wilk test), the Wilcoxon signed-rank test was used as a supplementary sensitivity analysis for paired comparisons (baseline vs. final follow-up) for both CRT and BRVA. For BRVA outcomes, eyes were further categorised into three groups: those gaining 1 or more lines (improvement of 0.1 logMAR or more), those losing 1 or more lines (worsening of 0.1 logMAR or more), and those with stable vision (change less than 0.1 logMAR). To account for potential correlation between bilateral eyes of the same patient, a linear mixed-effects model with a patient-level random intercept was used for the primary outcome analyses. Subgroup analysis was performed descriptively for the anti-VEGF monotherapy and anti-VEGF + rescue PDT groups. All statistical analyses were performed using Python (version 3.11.0). Linear mixed-effects models were fitted using the statsmodels library (version 0.14.0), while supplementary sensitivity analyses were performed using the SciPy library (version 1.18.0). A two-tailed *p*-value of less than 0.05 was considered statistically significant.

## 3. Results

### 3.1. Baseline Demographics and Clinical Characteristics

A total of 568 unique patients corresponding to 634 eyes were included in the analysis after splitting bilateral entries into individual eye-level observations. The mean age at presentation was 71.7 ± 8.8 years (range: 36–92 years), with a median of 72 years. The cohort was predominantly female, with 429 eyes (67.7%) from female patients and 205 eyes (32.3%) from male patients. Baseline clinical characteristics are summarised in Table 1. The mean follow-up duration was 11.5 ± 12.8 months (median: 6 months; Q1–Q3: 3–15 months; range: 1–48 months). A total of 201 eyes reached 12 months.

### 3.2. Treatment Burden

**Figure 1:**
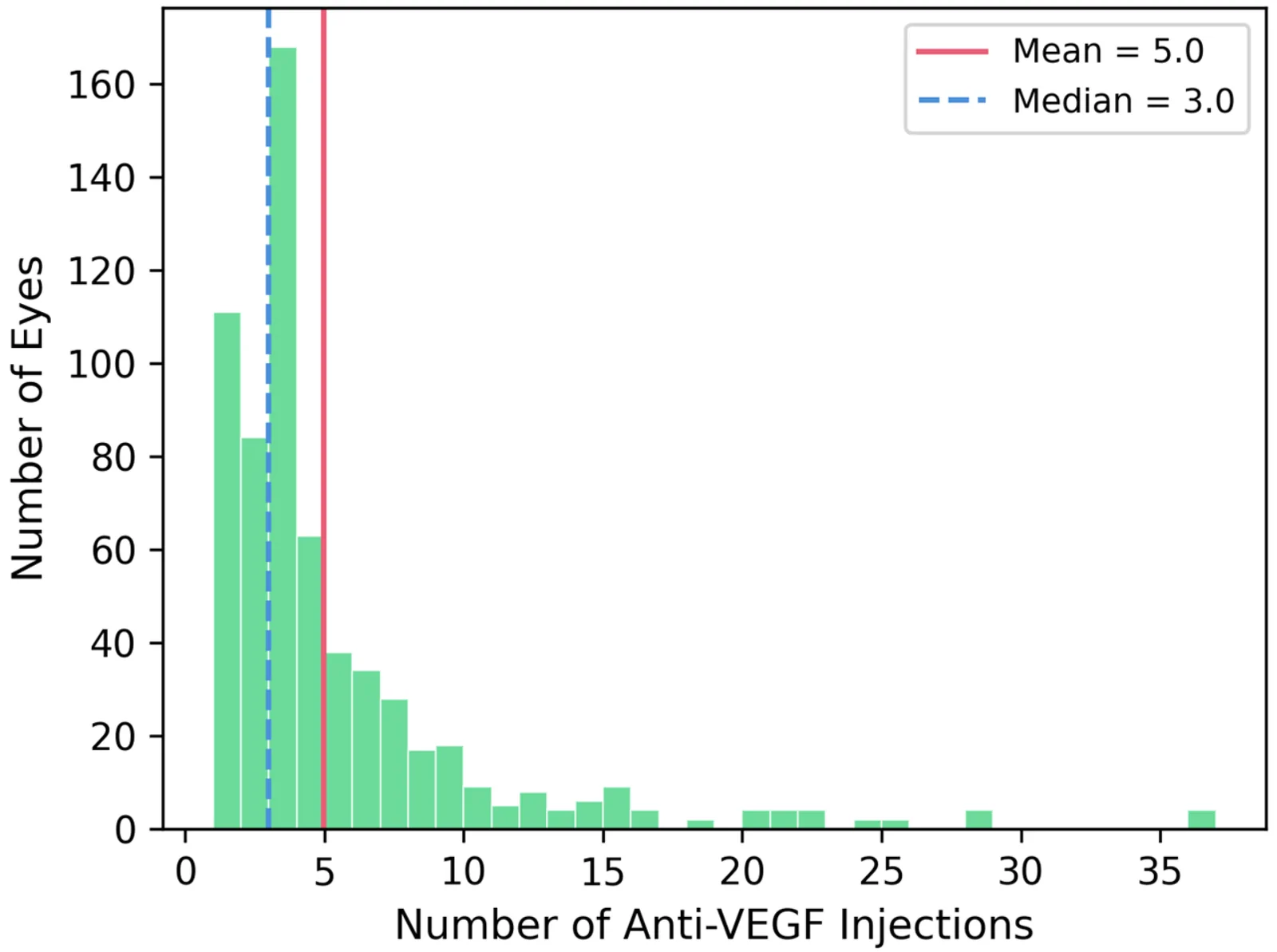
Distribution of the number of anti-VEGF injections per eye (*N* = 632 eyes). Red line: mean (5); blue dashed line: median (3).

The mean number of anti-VEGF injections per eye was 5 ± 5.3 (median: 3; range: 1–36). The distribution of injection burden was positively skewed, with the majority of eyes receiving fewer than 10 injections (Figure 1). This pattern reflects the heterogeneous follow-up durations and the treat-and-extend (T&E) or pro re nata (PRN) regimens commonly employed in routine clinical practice [23,24], as opposed to the fixed monthly or bimonthly dosing schedules used in clinical trials. The mean injection rate was 5.2 injections per eye-year.

311 eyes received bevacizumab, 279 received ranibizumab, and 44 received aflibercept. 65% of eyes received a loading dose of 3 consecutive monthly injections. The majority of treatments were administered using a PRN regimen (based on recurrence of fluid on OCT), while 12% were managed with a T&E approach. The main reasons for treatment discontinuation or loss to follow-up included patients’ perceived visual stability, logistical difficulties attending regular appointments and intervening systemic comorbidities. Agent switching occurred in 4% of cases.

### 3.3. Central Retinal Thickness

**Figure 2:**
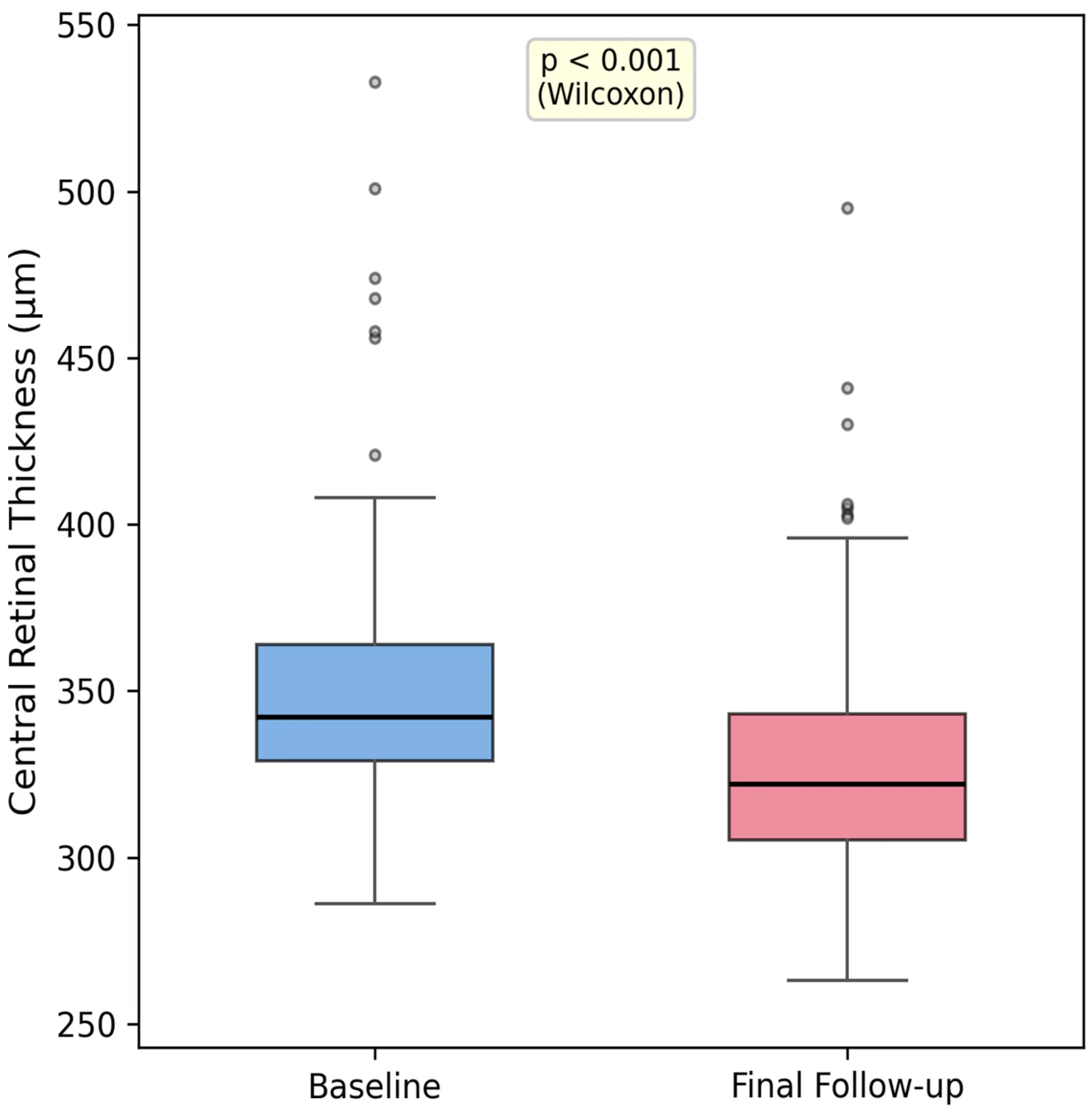
Comparison of central retinal thickness (CRT) at baseline and final follow-up (*N* = 634 eyes, *p* < 0.001). The horizontal line within each box represents the median.

**Figure 3:**
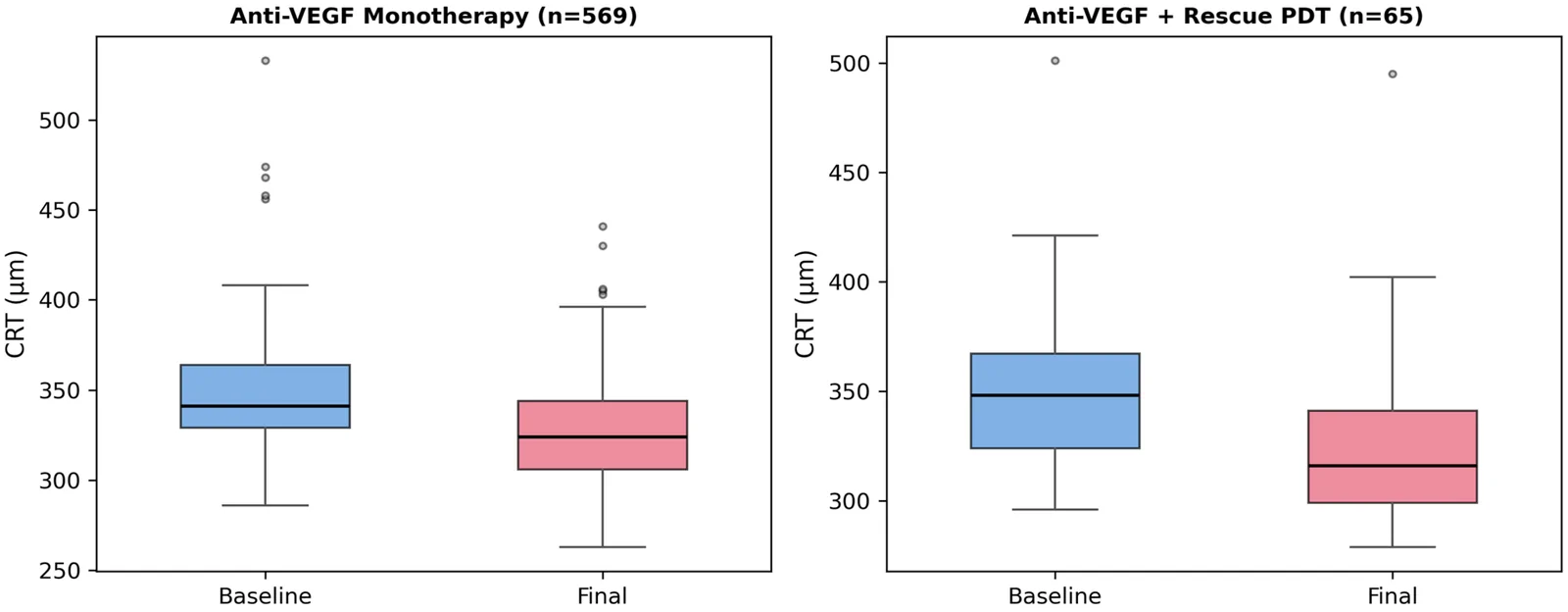
Central retinal thickness (CRT) reduction stratified by treatment subgroup: anti-VEGF monotherapy (**left**, *n* = 569) and anti-VEGF + rescue PDT (**right**, *n* = 65).

Using a linear mixed-effects model with a patient-level random intercept, the mean central retinal thickness decreased significantly from 347.3 ± 27.5 μm at baseline to 324.9 ± 28.8 μm at the final documented follow-up (estimate: −22.4 μm, 95% CI: −24.8 to −20.0, *p* < 0.001) (Table 2 and Figure 2). The median CRT decreased from 342 μm (IQR: 324–364) to 322 μm (IQR: 303–344). This anatomical response indicates that anti-VEGF therapy was associated with reducing retinal thickness, a surrogate marker for intraretinal and subretinal fluid resolution, across the treatment cohort. The CRT reduction was observed in both treatment subgroups (descriptive observation only), with the monotherapy group (*n* = 569) showing a mean reduction of 22.1 μm and the rescue PDT subgroup (*n* = 65) showing a mean reduction of 25 μm (Figure 3).

### 3.4. Best Recorded Visual Acuity

Using the mixed-effects model, mean BRVA in logMAR units improved from 0.98 ± 0.55 (Snellen ~20/190; ~35 letters) at baseline to 0.93 ± 0.56 (Snellen ~20/175; ~37.5 letters) at final follow-up (estimate: −0.05 logMAR, 95% CI: −0.08 to −0.02, *p* < 0.001), corresponding to a mean gain of approximately 2.5 ETDRS letters. While this change was statistically significant, its clinical significance is marginal, and the predominant functional outcome was visual stabilisation rather than meaningful improvement.

**Figure 4:**
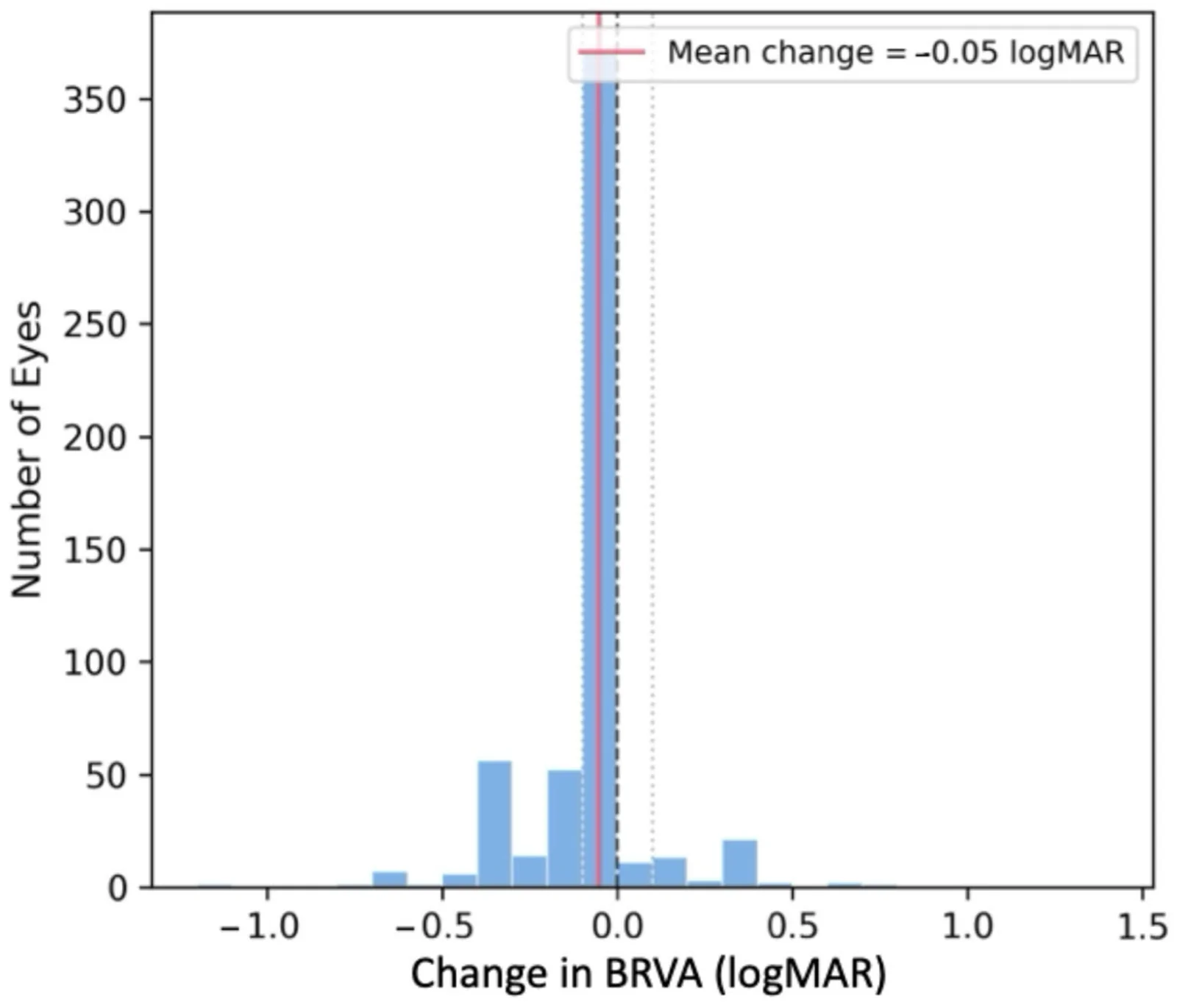
Distribution of best recorded visual acuity (BRVA) change in logMAR from baseline to final follow-up (*N* = 561 eyes). Negative values indicate improvement. The red line represents the mean change (−0.05 logMAR).

**Figure 5:**
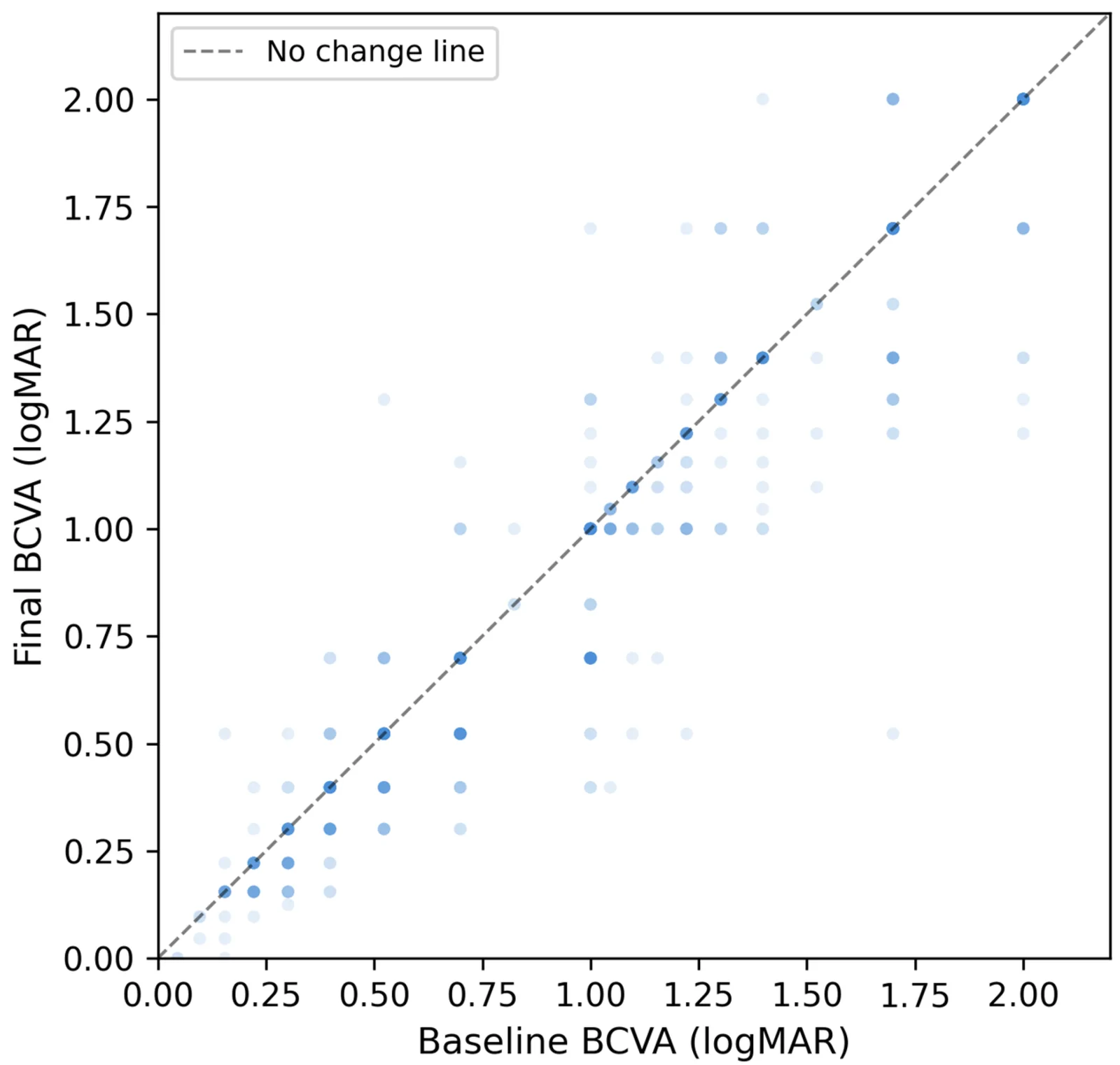
Scatter plot of baseline vs. final best recorded visual acuity (BRVA) in logMAR for 561 eyes. The dashed diagonal line represents no change.

When eyes were categorised by the magnitude of BRVA change, the majority demonstrated visual stability: 381 eyes (67.9%) had a BRVA change of less than 0.1 logMAR (stable), 138 eyes (24.6%) gained 1 or more ETDRS lines (improvement of 0.1 logMAR or more), and 42 eyes (7.5%) lost 1 or more ETDRS lines (worsening of 0.1 logMAR or more). A total of 8% of eyes gained ≥0.3 logMAR, 67.9% were stable (<0.1 logMAR), and 4% lost ≥0.3 logMAR. The distribution of BRVA change is illustrated in Figure 4, and the relationship between baseline and final BRVA is shown in Figure 5. Notably, the scatter plot demonstrates a strong clustering of data points along the identity line, particularly at baseline logMAR values between 0.7 and 1.3 (Snellen equivalent approximately 20/100 to 20/400), reflecting the predominantly poor baseline visual acuity in this cohort.

When stratified by initial anti-VEGF agent (Table 3), eyes receiving bevacizumab (49%) showed a mean CRT reduction of 22 μm and a mean BRVA change of −0.04 logMAR (~2 letters). Eyes receiving ranibizumab (44%) showed a mean CRT reduction of 22.8 μm and a mean BRVA change of −0.06 logMAR (~3 letters). Eyes receiving aflibercept (7%) showed a mean CRT reduction of 23 μm and a mean BRVA change of −0.08 logMAR (~4 letters). Outcomes were broadly comparable across agents, although the small sample size in the aflibercept group precludes formal comparative statistical conclusions.

### 3.5. Rescue Photodynamic Therapy Subgroup

A total of 65 eyes (10.3% of the cohort) received adjunctive rescue PDT with verteporfin (Visudyne) for persistent exudation despite a mean of 7.6 prior anti-VEGF injections (range: 2–24). PDT was performed according to the standard protocol at a mean of 8.5 months after treatment initiation. Following PDT, eyes received a mean of 2.1 additional anti-VEGF injections over a mean post-PDT follow-up of 3.2 months. The PDT subgroup showed a mean CRT reduction of 25 μm (from 348.6 ± 32 μm to 323.6 ± 35.4 μm) and a mean BRVA change of −0.05 logMAR (from 0.84 ± 0.64 logMAR at baseline to 0.78 ± 0.56 logMAR at final follow-up), corresponding to a mean gain of ~2.5 letters. The anatomical reduction was numerically similar to the 22.1 μm reduction observed in the anti-VEGF monotherapy group (descriptive only; not indicative of comparative effectiveness). Given significant confounding by indication, as this subgroup likely represented eyes with more refractory disease, this comparison is descriptive only and precludes conclusions regarding the incremental benefit of PDT in this setting.

## 4. Discussion

The principal finding of this real-world retrospective study is the dissociation between anatomical and functional outcomes in nAMD patients treated with intravitreal anti-VEGF therapy. While a statistically significant and clinically meaningful reduction in central retinal thickness was observed, the corresponding improvement in visual acuity was statistically significant but clinically marginal (mean gain of approximately 2.5 ETDRS letters). The overwhelming majority of eyes demonstrated visual stability rather than substantial improvement, and nearly one-quarter of eyes achieved a gain of 1 or more lines. This pattern of anatomical success coupled with limited functional recovery is consistent with a growing body of real-world evidence and reflects the intrinsic challenges of managing chronic nAMD in routine clinical settings.

The anatomical results of this study are broadly consistent with those reported in major clinical trials [7,8,9,15,16], although the magnitude of CRT reduction is somewhat smaller. The more modest CRT reduction observed in our cohort likely reflects the substantially lower treatment intensity, with a mean of only 5 anti-VEGF injections compared to the 12–24 injections typically administered over two years in trial protocols. This lower treatment intensity is a hallmark of real-world nAMD management and may be related to a combination of unmeasured factors, including heterogeneous follow-up durations, variable disease activity, and treatment discontinuation [19,20,21,22]. The dissociation between anatomical and functional outcomes in our cohort may reflect irreversible baseline structural damage, such as macular atrophy or fibrosis, that limits visual recovery despite successful fluid resolution.

The visual acuity outcomes in this cohort are similarly aligned with real-world evidence [7,8,9,10,11,12,13,14,15,16,17,18]. Several interrelated factors contribute to this disparity. First, patients in real-world settings often present later in the disease course with established atrophic changes, fibrosis, or chronic subretinal fluid accumulation [25,26] that limit the potential for visual recovery regardless of treatment response. The mean baseline logMAR of 0.98 in our cohort reflects this delayed presentation pattern. The mean final visual acuity of ~37.5 ETDRS letters in our cohort remains below the 70-letter (Snellen ~20/40) threshold required for driving in most countries [27], underscoring the functional consequences of delayed presentation and under-treatment in real-world settings.

Second, the mean of 5 injections per eye suggests significantly lower treatment intensity compared to clinical trial protocols. The median of only 3 injections further underscores the fact that a large proportion of eyes received very few injections, likely reflecting heterogeneous follow-up, variable disease activity, or treatment discontinuation. While adherence problems, healthcare system constraints, and delayed presentation are often cited as contributors to the efficacy–effectiveness gap [28,29], these factors were not directly measured in this study. Unmeasured confounding and heterogeneous follow-up may also explain these findings.

An important observation in our study was the comparable anatomical and functional outcomes between bevacizumab and ranibizumab. Real-world outcomes for bevacizumab are particularly valuable in the current rapidly evolving treatment landscape. With the recent introduction of ranibizumab biosimilars [30], faricimab [31], and high-dose aflibercept (8 mg) [32], cost-effectiveness analyses are increasingly critical for healthcare systems. In resource-constrained settings [33], where healthcare budgets limit the universal adoption of newer, more expensive therapies, bevacizumab remains a vital and cost-effective option. Our finding that bevacizumab yielded similar fluid resolution and visual stabilisation to ranibizumab reinforces its ongoing role in maximising treatment access and injection capacity for broader patient populations, even as the pharmacological landscape expands.

Verteporfin PDT was used as an adjunctive therapy [34] in 10.3% of eyes, presumably for persistent or recurrent exudation despite anti-VEGF monotherapy. Because this subgroup likely represented eyes with more refractory disease, the anatomical outcomes of this subgroup are descriptive only. Any numerical differences between subgroups must not be interpreted as incremental efficacy of PDT, as confounding by indication precludes such conclusions. The use of PDT as a rescue strategy reflects a real-world clinical approach to managing anti-VEGF-resistant [35] or tachyphylactic [36] cases. However, it is important to note that the PDT subgroup likely represents a more severe or treatment-resistant phenotype, and the observed anatomical improvement should be interpreted cautiously in the absence of a control group.

Several important limitations of this study must be acknowledged. First, the retrospective design introduces inherent biases, including selection bias, information bias from incomplete or inconsistently recorded data [37], and the inability to control for confounding variables [38] that were not captured in the clinical database. Second, the absence of standardised follow-up intervals is a major limitation. The “final documented follow-up” varied substantially between patients, ranging from a single post-injection visit to extended treatment courses. This heterogeneity precludes the analysis of outcomes at standardised time points (e.g., 12 months) and may introduce bias if eyes with longer follow-up represent a selected subgroup with different disease characteristics or treatment responses. Third, the study lacks detailed OCT biomarker data, including the presence or absence of intraretinal fluid, subretinal fluid, pigment epithelial detachment [39,40], and atrophic changes such as geographic atrophy or fibrotic scarring [25,26,41]. These biomarkers have been shown to be important predictors of visual outcomes and would have substantially enriched the analysis.

Additional limitations include the inability to assess treatment adherence patterns over time, the lack of data on adverse events, and the small number of cases with aflibercept injection, which precluded formal statistical comparisons between various anti-VEGF agents. The findings should therefore be interpreted as descriptive real-world observations rather than definitive efficacy data.

## 5. Conclusions

In this large single-centre real-world cohort of 634 eyes with neovascular AMD, intravitreal anti-VEGF therapy was associated with significant anatomical improvement as evidenced by a meaningful reduction in central retinal thickness. At the same time, functional outcomes were characterised predominantly by visual stabilisation rather than substantial gains. These findings underscore the gap between RCT efficacy and real-world effectiveness in nAMD management and highlight the need for strategies to increase treatment intensity, reduce treatment burden, adopt personalised treatment approaches, and identify patients who may benefit from alternative or adjunctive therapies.

## Figures and Tables

**Table 1:** Baseline demographic and clinical characteristics, *N* = 634 eyes.

Parameter	Value
Patient-level characteristics (*N* = 568 patients)	
Mean age, years (±SD)	71.7 ± 8.8
Age range, years	36–92
Female, *n* (%)	388 (68.3%)
Male, *n* (%)	180 (31.7%)
Eye-level characteristics (*N* = 634 eyes)	
Total eyes (from 568 patients)	634
Eyes from female patients, *n* (%)	429 (67.6%)
Eyes from male patients, *n* (%)	205 (32.4%)
Right eyes (OD), *n*	257
Left eyes (OS), *n*	377
Eyes with eccentric fixation, *n*	28
Eyes available for best recorded visual acuity (BRVA) analysis, *n*	561 *

* 28 eyes with eccentric fixation and 1 eye with no light perception were excluded from BRVA analysis. OD/OS imbalance is the actual clinical distribution recorded (not a parsing error).

**Table 2:** Summary of anatomical and functional outcomes using the primary mixed-effects model. Values are presented as mean ± SD.

Outcome Measure	Baseline	Final	Estimate (95% CI)	*p*-Value (Primary)	*p*-Value (Sensitivity) †
Central retinal thickness (CRT), µm (*N* = 634)	347.3 ± 27.5	324.9 ± 28.8	−22.4 (−24.8 to −20)	<0.001	<0.001
Best recorded visual acuity (BRVA), logMAR (*N* = 561)	0.98 ± 0.55	0.93 ± 0.56	−0.05 (−0.08 to −0.02)	<0.001	<0.001

† Sensitivity analysis performed using the Wilcoxon signed-rank test for paired comparisons.

**Table 3:** Anatomical and functional outcomes stratified by initial anti-VEGF agent.

Outcomes	Bevacizumab	Ranibizumab	Aflibercept	Overall Cohort
Eyes (*n*)	311	279	44	634
Baseline CRT (μm)	347.5 ± 27	346.8 ± 28	350.1 ± 26.5	347.3 ± 27.5
Final CRT (μm)	325.5 ± 28	324 ± 29.5	327.1 ± 29	324.9 ± 28.8
Mean CRT change (μm)	−22 ± 24.5	−22.8 ± 25	−23 ± 22	−22.4
Baseline BRVA (logMAR)	0.99 ± 0.55	0.96 ± 0.54	0.95 ± 0.5	0.98 ± 0.55
Final BRVA (logMAR)	0.95 ± 0.56	0.9 ± 0.55	0.87 ± 0.52	0.93 ± 0.56
Mean BRVA change (logMAR)	−0.04 ± 0.5	−0.06 ± 0.52	−0.08 ± 0.45	−0.05

## Data Availability

The original contributions presented in the study are included in the article. Further inquiries can be directed to the corresponding author.

## Abbreviations

The following abbreviations are used in this manuscript:

AMD	Age-related macular degeneration
anti-VEGF	Anti-vascular endothelial growth factor
BRVA	Best recorded visual acuity
CATT	Comparison of Age-Related Macular Degeneration Treatments Trials
CRT	Central retinal thickness
ETDRS	Early Treatment Diabetic Retinopathy Study
nAMD	Neovascular age-related macular degeneration
OCT	Optical coherence tomography
OD	Oculus dexter (right eye)
OS	Oculus sinister (left eye)
OU	Oculus uterque (both eyes)
PDT	Photodynamic therapy
PRN	Pro re nata
RCT	Randomised controlled trial
SD	Standard deviation
SD-OCT	Spectral-domain optical coherence tomography
T&E	Treat-and-extend
